# Development of Essential Oil Delivery Systems by ‘Click Chemistry’ Methods: Possible Ways to Manage Duchenne Muscular Dystrophy

**DOI:** 10.3390/ma16196537

**Published:** 2023-10-02

**Authors:** Greta Kaspute, Bharani Dharan Arunagiri, Rakshana Alexander, Arunas Ramanavicius, Urte Samukaite-Bubniene

**Affiliations:** 1 Department of Nanotechnology, State Research Institute Center for Physical Sciences and Technology (FTMC), Sauletekis av. 3, LT-10257 Vilnius, Lithuania; greta.kaspute@ftmc.lt; 2 Department of Physical Chemistry, Institute of Chemistry, Faculty of Chemistry and Geosciences, Vilnius University, Naugarduko Str. 24, LT-03225 Vilnius, Lithuania; bharani.dharan.arunagiri@stud.vdu.lt (B.D.A.); rakshana.alexander@chgf.stud.vu.lt (R.A.)

**Keywords:** essential oil, click chemistry, targeted drug delivery system, Duchenne muscular dystrophy, phytotherapy, muscle-wasting disease, safeguard, drug conjugate, muscular dystrophy, nanotechnology

## Abstract

Recently, rare diseases have received attention due to the need for improvement in diagnosed patients’ and their families’ lives. Duchenne muscular dystrophy (DMD) is a rare, severe, progressive, muscle-wasting disease. Today, the therapeutic standard for treating DMD is corticosteroids, which cause serious adverse side effects. Nutraceuticals, e.g., herbal extracts or essential oils (EOs), are possible active substances to develop new drug delivery systems to improve DMD patients’ lives. New drug delivery systems lead to new drug effects, improved safety and accuracy, and new therapies for rare diseases. Herbal extracts and EOs combined with click chemistry can lead to the development of safer treatments for DMD. In this review, we focus on the need for novel drug delivery systems using EOs as the therapy for DMD and the potential use of click chemistry for drug delivery systems. New EO complex drug delivery systems may offer a new approach for improving muscle conditions and mental health issues associated with DMD. However, further research should identify the potential of these systems in the context of DMD. In this review, we discuss possibilities for applying EOs to DMD before implementing expensive research in a theoretical way.

## 1. Introduction

In recent times, the need for improved and safer therapies for diseases has taken priority. Rare diseases (RD) are among those that have gotten recent spotlight due to the need to improve the lives of the patients diagnosed and their families. 

Duchenne muscular dystrophy (DMD) is one such disease, affecting approximately 1 in 3500 male births worldwide. As the disease progresses, muscle weakness and atrophy spread to affect the trunk and forearms and gradually progress to involve additional muscles of the body [1]. This disease is an X-linked myopathy caused by a mutation in the dystrophin gene, which leads to dystrophin function disruption. Because of that, there is a loose connection between the actin cytoskeleton and connective tissue, and muscle fibers are easily damaged during contraction. As a result, DMD patients are affected by chronic muscle damage, inflammation, and eventually muscle fiber replacement by fat and fibrotic tissue, resulting in a loss of muscle function [2]. 

Today, the glucocorticoids prednisone/prednisolone and deflazacort are the gold standard of care for the treatment of DMD. However, the therapeutic benefits of glucocorticoids are limited by several adverse effects associated with their long-term use, e.g., osteoporosis, obesity, short stature, delayed puberty, and adrenal insufficiency [3,4]. Additionally, DMD has also been treated with nonsteroidal anti-inflammatory drugs (NSAIDs) since they pose less severe side effects and focus on muscle regeneration. However, there is still a need for further NSAID effects and safety studies regarding muscle regeneration [3,5]. Another problem associated with DMD is that patients have various DMD side effects that need to be cured and monitored, e.g., cardiomyopathy, affected physical development, scoliosis, etc. Patients with DMD and female carriers of DMD are highly at risk for cardiomyopathy leading to heart failure. Because of that, angiotensin-converting enzyme inhibitors are widely used to tackle this. A recent study also showed that patients with DMD with mutations in the Dystrophin Dp116 coding region have less frequent cardiac dysfunction compared to others [6]. 

Recently, the development of novel drug delivery systems has become very important to seek new drug effects and improved safety and accuracy. One of the fields to find new molecules to work with is phytotherapy, which contains many medicinal herbs and ways to produce their medical effects. For example, EOs have been used as a medicine for centuries in diverse cultures, and when combined with novel methods such as click chemistry, they can be a breakthrough for developing safer therapies for rare diseases. In our hypothesis, using EOs as a complex drug delivery system might aid in improving muscle conditions in patients with DMD. The increase in complementary and alternative medicine (CAM) use for various diseases leads people to seek out CAM applications to treat neuromuscular disorders, such as DMD. In Canada, 20% of Duchenne caregivers report administering CAM to their child together with traditional treatment [7,8]. Although nutraceuticals cannot cure DMD, they can have significant potential as complementary therapies to reduce chronic inflammation or oxidative stress [8]. For example, according to scientific data, acupuncture and Traditional Chinese Medicine (TCM) improve patient outcomes for patients with DMD. Researchers from Zhengzhou Children’s Hospital combined acupuncture, far infrared therapy, TCM tuina massage, herbal medicine, and drug therapy into a protocolized regimen of care. The combined therapy delivered significant results, including reduced physical impairments and improvements in both walking and staircase climbing. In addition, significant reductions in inflammatory-related muscle enzyme secretions were achieved [9]. 

In this review, we focus on the need for novel drug delivery systems using EOs, which can be potentially used for the therapy of DMD. We also discuss the potential use of click chemistry for drug delivery systems, aiming to improve the lives of those diagnosed and possibly have less severe side effects than the existing drugs. In addition, in this review, we encourage the application of science-based data from EOs for DMD research. Moreover, we want to further discuss and share information about possible ways to combine multiple disciplines into a relevant one because that receives too little attention. It is important to theorize, hypothesize, and amplify the conversation at the international level about EOs application opportunities for DMD before implementing expensive research. 

## 2. Importance of New Drugs Development in Rare Diseases Field

**Figure 1:**
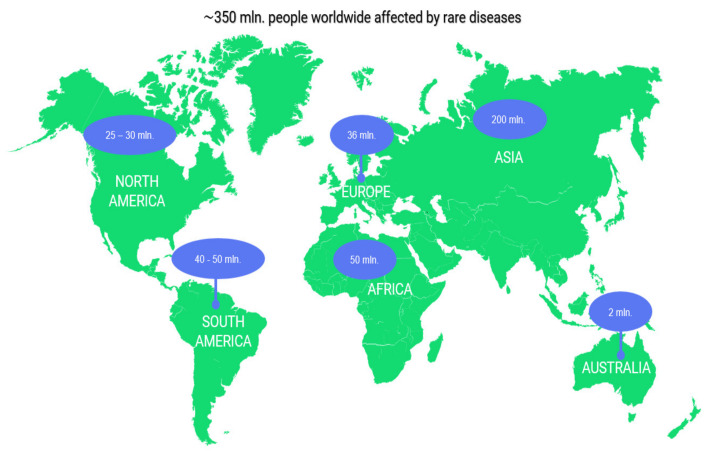
The number of rare diseases that affect people worldwide, by continent [11,14,15,16,17,18,19,20,21].

Many rare diseases are extreme manifestations of common diseases. Scientists have stated that research on rare diseases is critical for identifying cellular and molecular pathways that are linked to the development of other diseases [10]. These diseases affect about 350 million people worldwide; however, low prevalence is associated with many challenges, e.g., lack of knowledge and expertise (Figure 1). There are around 6–8 thousand rare diseases [11], which can be defined as rare when they affect 1 person per 2000 in Europe [12] or 1 person per 1650 in the US [13]. 

Most rare diseases (70%) are genetic, associated with chronic pathways, and can affect all organ systems [14,22]. Common diseases might also share many genetic pathways with RDs, whose identification would lead to the discovery of new targets important for the development of drugs [10]. Therefore, there is a need for better knowledge of RD epidemiology to determine the specific needs of rare disease patients, improve the development of novel therapies, and encourage the participation of rare disease patients in clinical trials [22]. 

Muscular dystrophies (MDs) are a heterogeneous group of genetic disorders and can be defined as the progressive loss of muscle strength and integrity. Currently, a molecular genetic approach, positional cloning, and candidate gene analysis have identified more than 30 genes responsible for MDs [23]. To add to that, neuromuscular disorders encompass a heterogeneous group of conditions that impair the function of muscles, motor neurons, peripheral nerves, and neuromuscular junctions [24]. 

**Figure 2:**
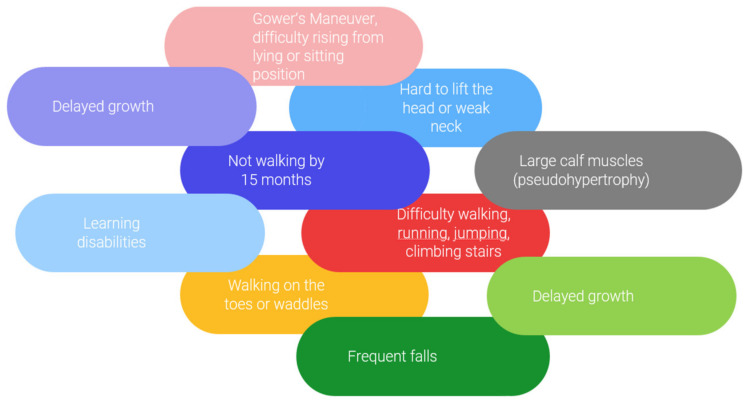
Signs and symptoms typically appear in Duchenne muscular dystrophy (DMD) in the early childhood phase [28,31].

One of the MDs is Duchenne muscular dystrophy, which is a severe, progressive, muscle-wasting disease caused by mutations in the encoding 427-kDa cytoskeletal protein dystrophin gene in muscle. The DMD leads to difficulties in movement, the need for assisted ventilation, and premature death [25,26]. It is a lethal pediatric disease, and currently there is no cure [27]. The average age of the first Duchenne signs is around 4 years old [28]. Mutations in the dystrophin gene lead to progressive muscle fiber degeneration and weakness, cardiomyopathy, and death in early adulthood [29,30]. Thus, patients are unable to carry out their daily activities and must use wheelchairs [30]. It is important to identify the first signs and symptoms of DMD early to control the disease. The main symptoms are associated with difficulty walking, jumping, running, climbing stairs, pain of muscle, pseudohypertrophy, delayed growth, learning disabilities, and others (Figure 2) [28,31]. 

The dystrophin gene is found in the X-chromosome and primarily affects males [29,32]. Thus, females are typically disease carriers. To add, less severe than Duchenne is Becker MD (BMD), when dystrophin is manufactured in a non-normal form or amount [25,33]. Individuals with BMD generally have mutations that maintain the open reading frame, allowing the production of dystrophin proteins with an internal deletion or duplication that can connect actin to the connective tissue, and thus are partially functional. As a result, patients with BMD often show a later onset and a slower disease progression. Because of phenotypic variation, patients are diagnosed in childhood, midlife, or later [2]. The dystrophin gene is important as it is structural in muscle and links the internal cytoskeleton to the extracellular matrix [25]. The frequency of DMD mutations may depend on geography and race and come as single or multi-exon deletions, especially between exons 45 and 55 and in the region between exon 3 and exon 19 of the dystrophin gene [34]. Drugs are used to disrupt translational fidelity by interacting with the ribosomal Ribonucleic acid(RNA) to translate the full-length dystrophin despite the presence of the premature stop codon. Gentamicin was the first aminoglycoside drug used for this type of approach; however, there is no updated data related to the clinical efficacy of this drug [34]. For example, Exondys 51 (Sarepta Therapeutics, Inc., Cambridge, MA, USA) was the first drug to obtain The United States Food and Drug Administration (FDA) accelerated approval as this approach uses antisense oligonucleotides (ASOs), which are designed to bind and modulate RNA functions through different mechanisms. In DMD patients whose mutations are amenable to exon 51 skipping, this treatment has increased dystrophin expression by 0.28% and by 0.93% after 48 and 180 weeks of treatment, respectively [34]. One more drug, Casimersen, also known as AMONDYS 45™, received accelerated approval from the FDA in February 2021 for the treatment of DMD patients amenable to exon 45 skipping [34]. 

Another problem related to the dystrophin gene is the onset of immunological reactions. Thus, there is a need for long-term delivery of the missing gene, or persistent gene correction, in most muscle fibers of a DMD patient. This leads to the need for multiple gene therapy injections [25]. Studying *mdx* mouse model showed that advances in viral delivery (e.g., functional dystrophin mini- and micro genes and gutted vectors with large insert capacity and lowered immunogenicity) improved muscle function and the expression of dystrophin [35]. To add to that, adeno-associated viral vector (AAV) gene therapy has the potential to treat the majority of the DMD patient population, regardless of genotype. This AAV therapy showed promising effectiveness results in preclinical trials and, thus, can be relatively safe in clinical ones too [36].

Moreover, stem cell (SC) therapy shows promising results. Vessel-associated fetal SC, ‘mesoangioblasts’, have been shown to provide widespread rescue of dystrophy in αsarcoglycan-negative mice after femoral artery delivery; moreover, lentiviral transduction of mesoangioblasts isolated from dystrophic mice before injection gave similarly optimistic results [25]. Histone deacetylase inhibitors (HDACi), such as valproic acid, phenylbutyrate, givinostat (ITF2357), and suberoylanilide hydroxamic acid, have been tested in DMD mouse models. Givinostat blocked the histone deacetylase enzyme and permitted the activation of the follistatin gene, leading to an increase in muscle mass. As a result, Givinostat prevented muscle degeneration, reducing the adipogenesis process and fibrotic tissue accumulation [34]. Some clinical data also showed successful human umbilical cord mesenchymal SCs [37] and myoblast transplantation in immunosuppressed patients, which led to dystrophin expression and stabilized muscle power [34]. 

To sum up, data shows that genomic editing in myogenic SCs, gene therapy combined with cell transplantation, and tissue engineering can have a positive impact on DMD therapy. These therapies are also applicable to other types of MDs [24]. 

## 3. Development of Novel Drug Delivery Systems

Drug delivery focuses on pharmaceutical compound administration to achieve therapeutic effects in humans or animals [38]. Thus, the science of drug delivery systems concentrates on the development of novel vehicles to improve drug delivery efficiency [39]. Developing novel drug delivery systems is an expensive and time-consuming process that leads to individual therapy development, dose titration, and therapeutic drug monitoring [38]. Despite being expensive, drug delivery systems have several advantages in contrast to traditional forms of drugs:

1. Minimized side effects on vital tissues because of their ability to be specifically transported to the place of action [40].
2. Opportunity to require lower doses of the drug because the accumulation of therapeutic compounds in the target site increases [40].
3. Cell-specific targeting is achieved by attaching drugs to designed carriers (nanoparticles, liposomes, etc.). [40]

Nanotechnology has made an impact on the development of nanoscale drug delivery devices while using important strategies to deliver conventional drugs, recombinant proteins, vaccines, and nucleotides [41]. New forms of drug delivery systems include liposomes, proliposomes, microspheres, gels, prodrugs, cyclodextrins, nanoparticles, exosomes, etc. [38,40].

Lipid- or polymer-based nanoparticles (NPs) can be used as advanced drug delivery systems to improve the pharmacological and therapeutic properties of parenterally administered drugs, e.g., several drug delivery system formulations with antifungal (Liposomal amphotericin B (AmBisome), Gilead Sciences, Inc., Foster City, CA, USA and Fujisawa Healthcare Inc., Elkridge, MD, USA) and anticancer (Liposomal doxorubicin (Myocet), Elan, Dublin, Ireland) drugs are approved for clinical use [42]. The advantages of NPs are their ability to be transferred into aerosols, stability, biocompatibility, targeting of specific receptors, and degradation within an acceptable period [38]. 

To add to that, in the pharmaceutical and cosmetic industries, lipid-based NPs and liposomes have been used to transport various molecules; also, liposomal encapsulation is used to achieve a stable platform for anti-cancer drug delivery [43]. However, this particular drug carrier still has not attained its full potential; there are still some aspects that need optimization. Their surfaces have to be modified to avoid uptake by the phagocytic cells of the reticuloendothelial system; drug release mechanisms need to be improved so that the drug is completely and exclusively deposited at the disease target site; and more efficient strategies for conjugating targeting moieties (such as peptides and antibodies) to liposomal surfaces are required. Many strategies rely heavily on uncontrolled chemical reactions, which frequently result in unwanted by-products. One of the most common methods involves the formation of amide bonds by the reaction of functionalized carboxylic acid groups with primary amines [44]. This method does not require prior ligand modification, which reduces the risk of ligand bioactivity loss; however, specificity is frequently lacking, resulting in an uncontrolled number of covalent bonds between the liposome and the targeting ligands. The reaction of thiol and maleimide functional groups to produce stable thioether bonds is a much more efficient conjugation method [45]. However, native thiol functional groups are either absent or present in insufficient amounts in many cases [46]. Scientists found the click reaction for easy in-situ surface modification of liposomes. During this study, alkyne-terminated 1,2-dioleoyl-sn-glycerol-3-phosphoethanolamine (DOPE), 1,2-dioleoyl-sn-glycerol-3-phosphocholine (DOPC), and a lissamine rhodamine derivative of DOPE (DOPE-LR) were sonicated together to form unilamellar liposomes with terminal alkyne groups. The vesicle solution was then treated with an azide-functionalized nitrobenzoxadiazole (NBD) derivative, N_3_-Lys(NBD)-NH_2_, and CuBr. The reaction was tracked in time using a method based on fluorescence resonance energy transfer, demonstrating that chemical modifications did occur at the surfaces of the liposomes. The reaction was completed in 4 h with no significant changes in liposome size [46,47].

One more research field is mesoporous silica NPs, which can be used to deliver therapeutic agents to treat various diseases. Mesoporous silica NPs can deliver small chemicals to large-sized peptides or proteins to fight against complex diseases. However, mesoporous silica NPs, because of their low bulk density, retaining mesoporous structure during downstream processing, and lack of sufficient in vivo studies, need further investigation [48]. 

Another promising alternative to synthetic drug delivery systems is an extracellular vesicle (EV)-based hybrid system. This is because drug delivery system hybridization with EVs leads to improved colloidal stability, enhanced cargo delivery and targeting profiles, and immuno-evasive properties [39]. Scientists found that, compared to EVs, exosome-like NPs derived from plants are source-widespread, cost-effective, and easy to obtain. Because of that, these NPs can be used to treat metabolic syndrome, colitis, cancer, hepatitis, etc. [49]. 

### 3.1. Importance of Novel Drug Delivery Systems Development for Herbal Medicine

Since ancient times, herbal medicine has been widely used all over the world because of its promising therapeutic effects and fewer side effects as compared to modern medicine [50]. Phytochemicals, which are substances produced by plants, show many biological activities, providing a scientific basis for using herbs in traditional medicine [51]. 

For some reasons, such as lack of scientific justification, processing difficulties (standardization, extraction), identification of herbal components, and herbal medicine, these were not considered important for novel formulations [50]. Another important issue is that phytoconstituents are highly sensitive to some environmental and physical factors, e.g., pH, oxygen, heat, etc. [52]. In addition, herbal medicine has low solubility, stability, and bioavailability [51,53]. 

Today, the drug delivery system used to administer herbal medicine lacks efficacy and has side effects from various compounds [50]. To add to that, novel drug delivery systems in herbal medicine should protect phytoconstituents from internal and external conditions [52]. Recently, nanotechnology-based herbal drug formulations have shown promising results in developing biocompatible, biodegradable drug delivery systems (lipids, polymers, and nanoemulsions), which increase the solubility, stability, bioavailability, and pharmacological activity of herbals [51,54,55]. For example, microparticulate drug delivery systems can be a choice for developing new drug delivery systems for herbal medicine. The main reasons are small size, targeted drug delivery, protection of constituents, and minimizing drug-related adverse reactions [52]. Another example could be nanogels, which are nanomedical products offering stability, drug loading capacity, strong penetration ability, biologic consistency, etc. New generation nanogels provide safer and more effective drug delivery and are able to be used in tissue engineering [55]. Other types of novel drug delivery systems: nanocapsules can be used to supply drugs to a particular region; nanoemulsions can be valuable for transdermal delivery systems because they are non-toxic and non-irritating, as well as improving the drug’s solubility and bioavailability [55]. 

Herbal extracts and EOs have been studied for their potential use in treating various medical conditions, including muscle disorders, while developing new drug delivery systems (Table 1). Some studies have suggested that certain EOs may have anti-inflammatory, antioxidant, and muscle-relaxing properties that could be beneficial in managing the symptoms of DMD. However, most studies on the use of EOs in DMD are preclinical studies on animal models; more research is needed to determine the effectiveness of EOs in humans with DMD. This review seeks to encourage the development of new drug delivery systems using herbal extracts, according to other successful studies on various diseases and health issues. 

#### 3.1.1. Solubility and Delivery of EOs in Blood

The EOs are frequently used in different industrial fields such as preservatives-aroma providers in food, fragrance-antioxidant-antiaging in cosmetics, cleaning, and as aromatherapeutic-phytotherapeutic agents in health [60,61]. Due to their physicochemical properties, such as high volatility and low aqueous solubility, EO delivery to target receptors can be challenging [62]. Encapsulating EOs in drug delivery systems offers benefits such as increased bioavailability, chemical stability, reduced toxicity, and targeted delivery [63]. Loading EOs into NPs can increase their affinity for targets, improve their penetration, and speed up their accumulation process in different cell types [64]. It has been shown that EOs increase skin penetration by interacting with the stratum and facilitating the passage of drugs through the skin [60,61]. Alveolar diffusion is the main pathway for molecular delivery, and some EO components have been reported as being soluble in water [65]. 

**Figure 3:**
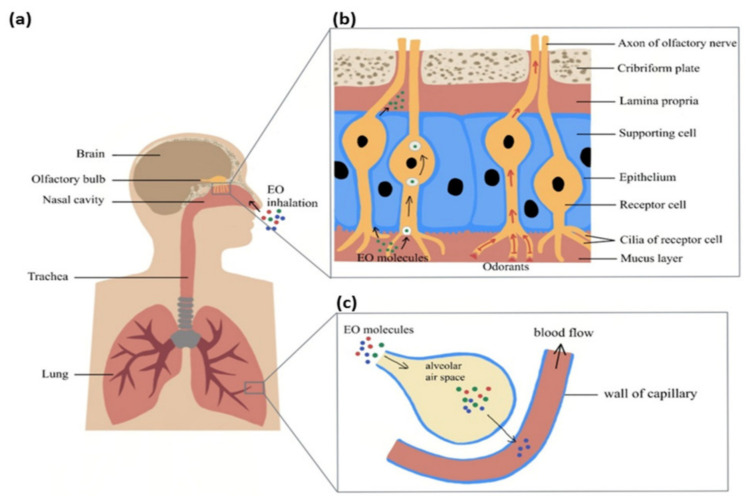
Mechanisms of molecules in oil delivery to the bloodstream. (**a**) Inhaled essential oil (EO) molecules traverse the nasal passage and access either the olfactory system or the respiratory system. (**b**) The pathway of EO molecules within the olfactory system. (**c**) A summary of how EO molecules enter the circulatory system [65]. Reprinted with MDPI permission.

The EOs are generally insoluble in water but soluble in alcohol, ether, and fixed oils [66]. Also, another research [67] indicate that essential oils penetrate only into the most superficial layers of the skin, furthermore, with a “filmogenic” mechanism improving the epidermal water balance [67]. The lipid solubility of EOs components allows these compounds to cross the blood-brain barrier and contact the fluids around the brain. However, taking EOs internally is not advised as it is the least effective way to absorb their therapeutic properties, and the oil ends up in the digestive tract, where it must pass through the stomach and small intestine before it reaches the bloodstream (Figure 3) [68]. 

Systems for delivering medications that cannot be dissolved in water have been replaced with oil-based ones to minimize their drawbacks. These methods boost the bioavailability of EOs, enhance their chemical stability, and lessen their volatility and toxicity. A vegetable-based oil, a gelling agent to maximize viscosity and heat stability, and a solubilizer to aid in the drug’s dissolution in the oleogel system make up the simple ingredients of the oleogel system [69]. The EOs have been discovered to interact with the stratum corneum to promote skin penetration [60]. The highly organized intracellular lipid structure in the stratum corneum is disrupted by EOs, which is the primary mechanism for enhancing the penetration of medicines by these substances [60]. Additional mechanisms include changing the stratum corneum membrane fluidity by means of EOs that cause the breaking of hydrogen bonds and changes in the condition, conformation, structure, and keratin of stratum corneum lipids and keratin [60].

After the inhalation of the NPs containing EOs, the molecules enter the lungs and reach the alveoli [63]. The NPs release their cargo of EO compounds into alveoli, whose membrane consists of a phospholipid bilayer, allowing the lipophilic EO molecules to readily diffuse across the membrane into the pulmonary capillaries. From there, the EOs compounds enter the bloodstream, where they can bind to carrier proteins or lipoproteins, facilitating their transport throughout the circulatory system to target tissues, cells, or organs. 

#### 3.1.2. EOs Constituents with NPs for Drug Delivery

Terpenes are the most common class of chemical compounds present in EOs. Many terpenes and EOs, however, are sensitive to environmental conditions, resulting in volatilization and chemical degradation. To overcome the chemical instability of some isolated terpenes and EOs, these compounds have been encapsulated in nanostructured systems (polymeric, lipids, or molecular complexes) (Table 2). Furthermore, because of its ability to improve bioavailability and allow for controlled drug release, nanoencapsulation may be of interest for pharmaceutical applications [70].

To develop new drug delivery systems to transport EOs, NPs can be used [64]. Various nanostructures, including liposomes, polymers, dendrimers, silicon or carbon materials, and magnetic nanoparticles, have been tested as carriers in drug delivery systems [40].

### 3.2. Application of Click Chemistry for DMD and EOs Molecule Development

Click chemistry (CC), as implied earlier, refers to strong linking reactions that are fast, have a high yield, are simple to perform, and are adaptable to joining various structures without the need for additional safeguards [46]. These properties make click chemistry an attractive strategy for the synthesis of drug conjugates for targeted drug delivery. In general, click chemistry can be used to create a wide variety of drug conjugates for targeted drug delivery by linking drugs to specific targeting moieties. This approach can improve the selectivity and efficacy of the drugs while minimizing their toxicity. However, it is important to note that while the use of click chemistry can greatly facilitate the synthesis of drug conjugates, it is not a panacea, and the efficacy of the conjugates still needs to be evaluated by in vitro and in vivo studies.

Bioorthogonal chemistry is a promising click chemistry that could be potentially used for this complex drug delivery system using EOs along with NPs and such since our goal is to work with living organisms. Bioorthogonal chemistry represents a class of high-yielding chemical reactions that proceed rapidly and selectively in biological environments without side reactions towards endogenous functional groups [74]. For example, in a recent report by Dos Santos Morais et al. [75], the dystrophin protein was modified using click chemistry/mass spectrometry, and small-angle neutron scattering methods. Most of the dystrophin protein is made up of a central domain composed of 24 spectrin-like coiled-coil repeats (R) [75]. The subdomains R1-3 and C-terminus (CT) were exclusively restricted to the muscle cell membrane [76]. It is known that the structure of the three first consecutive repeats 1–3 (R1–3) is a part of dystrophin known to physiologically interact with anionic membrane lipids. Moreover, the opening of the R1 coiled coil repeats when attached to membrane lipids, concluding that the sarcolemma membrane anchoring that occurs during the contraction/elongation process of muscles could be ensured by this coiled coil opening. Absence of sarcolemma leads to severe DMD. Understanding these structural changes may thus aid in the development of rationalized, shortened dystrophins for gene therapy [75]. 

Bioconjugation reactions are the most common pharmaceutical applications of click chemistry, excluding drug discovery and polymer chemistry. One reason for this is that 1,2,3-triazoles make ideal linkers. They are extremely water soluble, making in vivo administration much easier [46]. As one of the typical bioorthogonal reactions, the copper(I)-catalyzed azide-alkyne cycloaddition CuAAC reaction has been applied as a powerful synthetic tool for bioconjugations or coupling functional groups and molecules with fast kinetics and high yields under mild conditions [77,78].

Localized or targeted drug delivery systems have become the preferred choice of drug delivery, whose primary goals are to minimize the adverse effects of a drug and to address unmet and emerging needs for better patient compliance. Thus, modifications to the formulation or design of the drug molecules are not sufficient to maximize the clinical efficacy of targeted therapies. Given its mild reaction conditions and bio-friendly nature, click chemistry has been used for the synthesis of novel drug delivery systems, leading to products that can better meet the needs of individual patients [79].

#### 3.2.1. Polymer-Based Drug Delivery Systems

Polymeric drug delivery systems have shown the potential to treat a variety of diseases and are particularly effective against those with enhanced permeability and retention effects. Polymer chemistry has benefited from the simplicity and versatility of click chemistry (CC), and a variety of materials have been prepared, including terminal- and pendant-functional polymers; micelles; block co-polymers; and complex structures such as graft, star, brush, dendritic polymers, gels, polymersomes, and polymeric nanoparticles [79]. Polymers have played an integral role in the advancement of drug delivery technology by providing controlled release of therapeutic agents in constant doses over long periods, cyclic dosage, and effective release of both hydrophilic and hydrophobic drugs [80].

The polymers for drug delivery are logically classified based on the following characteristics:

1. Origin: synthetic, natural, or a combination of both [81].
2. Chemical nature: polyester, polyanhydride, protein-based, cellulose derivatives, etc. [81].
3. Backbone stability: biodegradable or nonbiodegradable [81].
4. Solubility: hydrophilic or hydrophobic in nature [81].

There are several types of polymer-based drug delivery systems, including:

1. Microspheres. These are small particles made of a polymer that can encapsulate drugs. They can be designed to release drugs over a period, making them useful for sustained release applications [82,83].
2. NPs are a wide class of materials that have one dimension less than 100 nm [84]. These particles can be used to improve the solubility and bioavailability of drugs [85,86], and they can also be designed to enhance drug release [87].
3. Hydrogels. These are three-dimensional networks of polymers that can absorb water and swell, making them useful for delivering drugs in a controlled manner. Stimuli-responsive hydrogels, also called “smart hydrogels,” are attractive for modern drug formulations because they can release drugs in response to external stimuli [88,89].
4. Polymeric Micelles: Polymeric micelles are nano-sized assemblies formed by amphiphilic block copolymers in dilute solution when the concentration of a polymer is increased. These self-assembling polymers can be used to encapsulate hydrophobic drugs and control their release profile in response to stimuli such as pH, enzymes, and temperature [90,91].
5. Polymer conjugates: Polymer conjugates are a type of drug delivery system that involves the use of polymers to facilitate the controlled release of therapeutic agents [80,92]. Polymeric drug delivery systems have been developed using both natural and synthetic polymers. Natural polymers such as arginine, chitosan, dextrin, polysaccharides, poly (glycolic acid), poly (lactic acid), and hyaluronic acid have been used in these systems [93]. Synthetic polymers such as dendrimers and dendron-polymer conjugates have also been used to create nanosized drug delivery vehicles [94].

All these polymer-based drug delivery systems have advantages and disadvantages, and the choice of the appropriate system depends on the characteristics of the drug and the target delivery site (Table 3). 

Williams et al. [109] introduced the idea that antisense oligonucleotides containing 2-O-methyl modifications can be developed into medications to treat DMD. The absence of efficient carriers, however, makes antisense oligonucleotides (ASO)-based methods difficult to use to transport ASOs to myonuclei. The study showed that cationic poly(ethylene imine) and polyethylene glycol copolymers could improve ASO transfection in *mdx* mice’s skeletal muscle. At 3 weeks after injection, ASO complexed with low Mw PEI2000 (PEG550) copolymers resulted in a widespread distribution of dystrophin-positive fibers with no apparent cytotoxicity in the tibialis anterior (TA) muscles of *mdx* mice. Overall, compared to ASO alone, injections of these low Mw polyplexes produced roughly six times as many dystrophin-positive fibers, which formed 250-nm aggregate particles [109]. Although this example is not of herbal medicine, there is an opportunity to implement similar research using EOs.

#### 3.2.2. Thiol-Ene Click Chemistry

The advantage of thiol-ene click chemistry is that it allows for the formation of covalent bonds between thiol and alkene groups in a highly selective, efficient, and mild manner. This reaction is widely used in the fields of materials science, organic synthesis, and biotechnology. In biotechnology, the thiol-ene click reaction is used to create new peptide and protein conjugates, as well as to link biomolecules to surfaces and nanoparticles. Thiol-ene click chemistry has been used in a range of applications in materials science, including the modification of the physical and chemical properties of surfaces and interfaces [110], macrocyclization, glycosylation, and peptide science [111], and stimuli-responsive materials [112]. In organic synthesis, it is used to synthesize new compounds with specific properties, such as fluorescent compounds [113] and bioactive compounds.

Thiol-ene click chemistry can potentially be used in the field of EOs, as it allows for the formation of covalent bonds between thiol compounds and alkene compounds. This could be used to create new EO compounds or to modify the properties of existing ones [114,115]. The thiol-ene click reaction can be used to create polymers, which are long chains of repeating molecules. These polymers can be used to deliver EOs, which are natural compounds found in plants that have medicinal properties. In the context of drug delivery for DMD, thiol-ene polymerization can be used to develop a carrier for EOs that can target the muscles affected by DMD, potentially improving the effectiveness of treatment. For example, EOs of lavender, lemongrass, thyme, and coconut can be modified using the thiol-ene click reaction to improve the positive impact on muscular pain, stress reduction, nausea, and inflammation.

#### 3.2.3. Diels-Alder Click (DA) Reaction

The DA cycloaddition is one of the metal-free click reactions; it is one of the most useful reactions in synthetic organic chemistry and material design. The DA reaction is a [4 + 2] cycloaddition of a conjugated diene and a substituted alkene (also termed dienophile), during which six π-electrons rearrange to form a cyclic, six-membered product. If both the diene and the dienophile are part of the same molecule, the DA reaction is usually referred to as an intramolecular DA cycloaddition [116]. The DA reaction has several variations based on substitution position and materials being coupled, which can affect the temperature threshold for and rate of retro reaction reversal. As a result, this reaction provides a straightforward coupling reaction for active ingredients with variable release [117]. 

Additionally, DA click chemistry has been used to synthesize monomers and polymers from plant oils [118], as well as highly efficient electro-optic polymers [119]. It is also regarded as a useful strategy in organic and macromolecular syntheses [120]. A variety of polymers have been used in DA click chemistry for EOs in drug delivery systems. One example is polyethylene glycol, which is a biocompatible and biodegradable polymer that can be used as a carrier for drugs [121]. Other polymers, including, Poly (vinyl alcohol), poly(vinylpyrrolidone), and poly(amidoamine), have been used as carriers for drugs [108,122].

#### 3.2.4. Copper-Catalysed Azide-Alkyne Cycloaddition (CuAAC)

The CuAAC is a widely used, reliable, and straightforward way for making covalent connections between building blocks containing various functional groups. This reaction is catalyzed by copper acetylide complexes that feature high activity even at low temperatures [123]. This reaction has been used in organic synthesis, medicinal chemistry, surface and polymer chemistry, and bioconjugation applications [124]. It is a 1,3-dipolar cycloaddition between an azide and a terminal or internal alkyne to give a 1,2,3-triazole. The CuAAC reaction region specifically produces 1,4-disubstituted-1,2,3-triazole molecules. This heterocycle formation chemistry has high tolerance to reaction conditions and substrate structures [125]. The reaction is also known as the azide-alkyne Huisgen cycloaddition.

Additionally, the CuAAC reaction has been used in peptide-based drug design [126]. It has also been used in polymer-based drug delivery systems, such as a polymer based on dynamic imine bonds [127]. To add, it has been applied to dendrimer synthesis and modification of the Hüisgen [2 + 3] cycloaddition between an alkyne and an azide catalyzed by copper under mild conditions [128]. Finally, a set of natural deep eutectic solvents have been investigated in CuAAC for use in EOs [129]. CuAAC has also been used to conjugate molecules to quantum dots for use in drug delivery systems [130]. 

This reaction is the cornerstone of “click” chemistry and has been used in a variety of applications, including the synthesis of EOs [129]. For example, CuAAC has been used to catalytically oxidize sustainable raw materials such as unsaturated fats and oils or fatty acids and their esters to produce biobased, high-value products [131]. The reaction is catalyzed by copper species and can be run in batches with P7 loaded with Cu(I) or Cu(II) salts [132]. 

Overall, CuAAC can be used in the synthesis of EOs and other biobased products. Various types of EOs have been used in combination with the CuAAC reaction. Some examples include:

- Terpenes: EOs such as limonene, pinene, and myrcene, which are rich in terpenes, have been used as the starting materials for the azide component of the reaction. Terpenes are a large and diverse class of natural products that are widely distributed in the plant kingdom [133]. One way that terpenes have been used in the CuAAC reaction is by derivatizing them with an azide-containing compound. This can be conducted by reacting the terpene with a reagent such as sodium azide or an azide-containing linker molecule. The resulting azide-derivatized terpene can then be reacted with an alkyne-containing compound in the presence of a copper catalyst to form the 1,4-disubstituted 1,2,3-triazole product [134]. Another way that terpenes have been used in the CuAAC reaction is by incorporating them directly into the alkyne component of the reaction. This can be conducted by synthesizing terpene-containing alkyne compounds, such as those with a terminal alkyne group, and then reacting these compounds with an azide-containing compound in the presence of a copper catalyst [124].
- Phenylpropanoids: EOs such as eugenol and thymol, which are rich in phenylpropanoids, have also been used as starting materials for the azide component of the reaction. Phenylpropanoids are a large class of naturally occurring compounds that are derived from phenylalanine and have a wide range of biological activities [135]. The CuAAC reaction is a widely used method for the efficient and selective formation of 1,4-disubstituted 1,2,3-triazoles. In the case of phenylpropanoids, researchers use this reaction to couple azide-functionalized phenylpropanoids with alkyne-functionalized phenylpropanoids, resulting in the formation of a 1,4-disubstituted 1,2,3-triazole product. This reaction typically requires the use of a copper (I) catalyst, such as copper (I) chloride, and is typically carried out in the presence of a ligand, such as 1,10-phenanthroline, to optimize the reaction conditions. The reaction is typically carried out in a polar solvent, such as dimethylformamide or dimethyl sulfoxide, at room temperature or slightly elevated temperatures [136,137].
- Alcohols: EOs such as linalool, geraniol, and citronellol, which are rich in alcohols, have also been used as starting materials for the azide component of the reaction. These EOs are known for their pleasant fragrances and are widely used in the perfumery and cosmetic industries [138]. The functionalization of these alcohol-rich EOs can be carried out by various methods, such as through the use of different reagents. For example, to functionalize linalool with an azide group, researchers can use *N*-hydroxysuccinimide and aryl azides or alkyl azides to introduce the azide group. Similarly, to functionalize geraniol with the alkyne group, researchers can use terminal alkynes and palladium catalysts to introduce the alkyne group. These functionalized EOs can be used in CuAAC reactions with other functionalized EOs or other molecules to produce a range of products, such as 1,4-disubstituted 1,2,3-triazoles [134]. It is important to note that reaction conditions such as solvent, temperature, and the concentration of reactants and catalysts play a crucial role in the efficiency of the reaction. Researchers often optimize these conditions to achieve high yields and selectivity for their desired products [134].

#### 3.2.5. Strain-Promoted Azide-Alkyne Cycloaddition (SPAAC)

The SPAAC is a bioorthogonal reaction involving the cycloaddition of a cyclic alkyne and an organic azide, leading to an aromatic triazole [139]. These highly efficient reactions that enable the assembly of molecules into complex structures have driven extensive progress in synthetic chemistry. In particular, reactions that occur under mild conditions and in benign solvents while producing no by-products and rapidly reaching completion are attracting significant attention. Amongst these, the SPAAC, involving various cyclooctyne derivatives reacting with azide-bearing molecules, has gained extensive popularity in organic synthesis and bioorthogonal chemistry. This reaction has also recently gained momentum in polymer chemistry, where it has been used to decorate, link, crosslink, and even prepare polymer chains [140]. 

The reactants for SPAAC are typically cyclooctynes and azides, which can be used to selectively modify biomolecules and living cells [139]. The reaction rate is influenced by the structure of the azide, with more reactive cycloalkynes being sought after in order to accelerate the process [141]. Cu-free click chemistry is another term for SPAAC, which utilizes a pair of reagents—cyclooctynes and azides—that exclusively and rapidly react with each other under mild conditions [142]. 

To add to that, SPAAC is a metal-free alternative to CuAAC and has been used in the design of polymer-based drug delivery systems [143,144]. SPAAC has been used to functionalize poly(amide)-based dendrons and dendrimers with poly(ethylene glycol) (PEG) chains under mild reaction conditions [144]. It has also been used to synthesize nanogels for sustained drug release [145] and pH-sensitive nanoscale drug delivery systems. An in vitro study with MCF-7 cells showed enhanced drug release at an acidic pH value compared to a neutral pH value. The profile of in vitro drug release exhibited enhanced drug release at acidic pH (pH = 5.3) compared with neutral pH (pH = 7.4). The results of the MTT (3-(4, 5-dimethylthiazol-2)-2, 5-diphenyltetrazolium bromide) assay showed that the amount of Doxorubicin (DOX) required for MCF-7 cells was reduced after DOX molecules were prepared into a polymer prodrug, which was conducive to reducing side effects under the premise of ensuring inhibitory effect [146]. Although this example is not of herbal medicine, there is an opportunity to implement similar research using EOs.

To sum up, click chemistry is an attractive strategy to synthesize drug conjugates for targeted drug delivery and to improve the selectivity and efficacy of the drugs while minimizing their toxicity. Although click chemistry can greatly facilitate the synthesis of drug conjugates, the efficacy of the conjugates still needs pre-clinical investigation. Click chemistry includes polymer-based drug delivery systems (microspheres, NPs, hydrogels, etc.); thiol-ene reactions to form covalent bonds between thiol and alkene compounds to modify EOs; DA reactions to produce monomers and polymers from plant oils; CuAAC for various EOs containing terpenes, alcohols, and phenylpropanoids; and SPAAC to develop polymer-based drug delivery systems.

## 4. Conclusions

In conclusion, the development of new EO complex drug delivery systems using click chemistry methods may offer a new approach for improving muscle conditions and mental health issues associated with DMD. These systems can potentially improve the bioavailability and effectiveness of EOs, which have been traditionally used in aromatherapy to alleviate symptoms of anxiety and depression. Currently, corticosteroids are the therapeutic standard for treating DMD, which can prolong ambulation and muscle function and cause serious adverse side effects. If a nutraceutical, e.g., herbal extracts or EOs, could produce similar therapeutic benefits to corticosteroids without adverse side effects, it could provide many DMD patients with an improved quality of life as well as reduce costs associated with recurrent hospital visits to monitor and treat corticosteroid-induced side effects [8]. However, it is important to note that further research is needed to fully understand the potential of these systems and to optimize their use for managing mental health in the context of DMD. This review is written to encourage the application of scientific knowledge by EOs to DMD research. Today, patients use complementary, alternative, and integrative medicine for various diseases, including muscular health issues. Because of that, it is important to theorize and hypothesize about the possibilities of applying EOs to DMD before implementing expensive research. 

## Figures and Tables

**Table 1:** Examples of drug delivery system development using essential oils (EOs).

Essential Oil	Drug Delivery Systems	Results	Reference
Zedoary (*Curcuma zedoaria*) oil	Self-nanoemulsifying drug delivery system	The active components remained stable in the optimized drug delivery system stored at 25 °C for at least 12 months. Oral administered Zedoary turmeric oil drug delivery system in rats. The area under the curve and maximum concentration of germacrone (GM), a representative bioactive marker of Zedoary turmeric oil, increased by 1.7-fold and 2.5-fold, respectively, compared with the unformulated Zedoary turmeric oil.	[56]
Cubeb (*Piper cubeba*) oil	Self-nanoemulsifying drug delivery system	Research was made to compare Cubeb (*Piper cubeba*) oil and standard antibiotic/gentamicin activity in female Wistar rats. Histopathological examinations of optimized drug delivery system treated animals showed no signs of inflammatory cells, which indicated that the prepared drug delivery system was safe and nontoxic to rats.	[57]
Ginger (*Zingiber officinale*) oil	Ginger EO was used along with lecithin (GL), cholesterol, and span 80 to fabricate nano-lipids (GL nano-lipids) using a thin-film method.	Due to the richness of shogaol-6 and other active compounds in ginger oil, the GL nano-lipid was endowed with intrinsic antibacterial activity. The sulforhodamine B (SRB) assay and live/dead imaging revealed the excellent biocompatibility of GL nano-lipids. GL nano-lipids were capable of carrying hydrophobic compounds such as curcumin and performed a pH-dependent release profile.	[58]
Oregano (*Origanum vulgare*) oil	Polymeric micelles drug delivery system as a possible non-invasive approach for the management of skin tags	The minimal inhibitory concentrations of the tested EO were similar to those obtained for the formulation: lower (2.5 µg/mL) for yeast and higher (40–80 µg/mL) for Gram-negative bacilli. EO-PbH decreased HaCaT cell migration and proliferation and elicited a cytotoxic and pro-apoptotic effect in a dose- and time-dependent manner. No harmful effect on the viability of dendritic cells was detected following the incubation with different concentrations (0–200 µg/mL) of the 5% formulation. Treatment of inflammatory dendritic cells (+ lipopolysaccharide (LPS)) indicated a decrease in cytokine production of IL-6, TNF-α, and IL-23 but no significant effect on IL-10 in any of the tested concentrations.	[59]
Bitter orange (*Citrus aurantium*) oil	Anti-inflammatory drug delivery system in Duchenne muscular dystrophy patients	Citrus has been proven to present anti-inflammatory action, however; components isolated from citrus still must be tested before their anti-inflammatory action on the striated muscle is ruled out.	[5]

**Table 2:** Examples of terpene encapsulation methods with nanoparticles (NPs).

Terpene	Method of Encapsulation	Importance	In Vivo Results	Reference
Thymol	Nanostructured Lipid Carriers (NLCs)	Thymol is an antimicrobial, antioxidant, and antiseptic compound with potential applications in wound healing and inflammation management.	Anti-inflammatory effects and improved healing in psoriasis were shown while using the cutaneous acute inflammation model induced by croton oil in BALB/c mice.	[71]
Triptolide	Solid Lipid NPs (SLNs)	The anti-inflammatory effect is over two-fold higher than that of conventional topological (TP) hydrogel.	Efficient transdermal delivery and anti-inflammatory activity while using the carrageenan induced edema mouse model.	[72]
Achyrocline satureioides EO	Achyrocline satureioides loaded nanocapsules (AS-NC) treatment	Natural compounds with antioxidant and free radical scavenging properties, such as Achyrocline satureioides EO loaded in nanocapsules (AS-NC), may be an important approach to reducing cardiac damage.	Protected against oxidative stress in the cardiac tissue of Wister’s rats.	[73]

**Table 3:** Advantages and disadvantages of different types of polymer-based drug delivery systems.

Type of Polymer-Based Drug Delivery Systems	Characteristics	Advantages	Disadvantages	Examples
Microspheres	Small polymer particles that encapsulate drugs	Improved efficacy and reduced toxicity [95].	Limited targeting capabilities (microspheres tend to migrate away from the injection site and lead to potential risks such as embolism) [96].	Sustained release applications [97].
NPs	Similar to microspheres but smaller (typically around 100 nm)	Improves the solubility and bioavailability of drugs [98].	Nanoparticles may not release drugs in concentrations high enough to result in cell death [99].	Targeting specific cells or tissues in the body [100].
Hydrogels	3D networks of polymers that absorb water and swell	Stimuli responsiveness, biocompatibility, high drug loading, and dependence of swellability on diffusivity of water [101].	Limited targeting capabilities (hydrogels are relatively insensitive and inadequate for low concentration samples, usually limited at around 0.1–0.2 mg/mL) [102].	Release drugs in response to changes in temperature, pH, or other stimuli [103,104].
Polymeric Micelles	Spherical structures formed by the self-assembly of amphiphilic polymers	Solubilize hydrophobic drugs and improve bioavailability [105].	Slow dissociation rates, which can limit the duration of drug release [106].	Core-crosslinked polymeric micelles with controlled release of covalently entrapped doxorubicin [107].
Polymer Conjugates	Drug covalently attached to a polymer	Improves pharmacokinetics and pharmacodynamics [80].	Low drug loading due to conjugation at the end [108].	Polymer-drug conjugates have a range of therapeutic applications [108].

## Data Availability

Not applicable.
